# American Dental Convention

**Published:** 1861-10

**Authors:** Geo. T. Barker


### Proceedings of Societies.

### AMERICAN DENTAL CONVENTION.

REPORTED BY GEO. T. BARKER, D. D. S.

THE Convention was called to order on Tuesday morning, August 6, 1861, at 11 o'clock, in the Music Hall, New Haven, Connecticut.

The President, Dr. T. L. Buckingham, in the chair.

The minutes of the last session were read by the Recording Secretary, Dr. B. W. Franklin, and after slight amendment, were approved.

A discussion relative to the admission of members ensued.

Dr. Rogers, of Utica, N. Y., suggested the issuing of tickets to members by the Treasurer on payment of their subscription, and that only those holding tickets be admitted on the floor.

Dr. Franklin opposed having tickets, and advocated opening the hall free to all who chose to attend. He wished the public, men, women, and children, would come in and fill the galleries. and so be educated in this most important subject.

Dr. Roberts, of New York, moved that all who wish to become members should sign their names on the roll and pay their subscription, which motion was carried.

The following gentlemen enrolled their names and residences, also paying the required assessment :

CALIFORNIA.—S. Crossett, North San Juan.

CONNECTICUT.—Samuel Mallett, I. Woolworth, H. J. Stevens, A. B. Smith, J. H. Smith, J. T. Metcalf, New Haven ; J. B. Snow, D. H. Porter, H. L. Sage, C. Merritt, Bridgeport; L. Parmele, J. S. McManus, E. E. Crofoot, Hartford; A. B. Welton, Milford; A. W. Allen, W. W. Clapp, Norwich; A. Hill, Norwalk; J. A. Pelton, Middletown; T. S. Scranton, Madison.

MAINE.—J. M. Nevins, Bucksport.

MASSACHUSETTS.—J. Beals, Greenfield; I. J. Wetherbee, Boston; W. W. Rice, Great Barrington; W. L. Bowdoin, Salem; Thos. Palmer, Fitchburg; S. G. Henry, Westboro; H. M. Miller, Westfield; F. Searle, Springfield.

NEW JERSEY.—S. J. W. Neal, Camden; C. S. Stockton, Mount Holly.

NEW YORK.—T. H. Burras, D. A. Jarvis, W. B. Roberts, A. McIlroy, C. S. Miles, B. W. Franklin, John Allen, A. Jones, W. H. Allen, G. H. Perine, New York City; Geo. E. Hayes, B. T. Whitney, Buffalo; A. N Priest, L. W. Rogers, Utica; W. B. Heard, W. W. Starr, Brooklyn; S. Mapes, Fishkill; M. Frank, Cortlandt; T. D. Evans, New York Mills; J. C. Smithe, Millerton; H. N. Fenn, Rochester; H. F. Smith, Pine Plains; W. S. Elliott, Sag Harbor; L. W. Sutton, Greenport; A. H. Brockway, Chittenango.

OHIO.—W. H. Atkinson, Chas. Butler, Cleveland.

PENNSYLVANIA.—T. L. Buckingham, S. S. White, J. D. White, Geo. T. Barker, J. L. Asay, A. Merritt Asay, J. R. McCurdy, Philadelphia; James Fleming, Harrisburg.

VERMONT.—M. Tefft, West Poultney; J. N. Scranton, Bennington.

The Treasurer, Dr. A. N. Priest, of Utica, N. Y., made his report, which was referred to an Auditing Committee, consisting of Drs. Burras and John Allen, and upon their recommendation was accepted.

Dr. F. Searle, of Springfield, Mass., as one of the Executive Committee, reported that Dr. F. Y. Clark, of Savannah, Ga., having seceded, they had added Dr. B. T. Whitney, of Buffalo, to the Committee.

Dr. Whitney stated that Dr. Clark retained the programme in his possession which had been adopted by the committee, and not being able to get it, a new one had to be prepared, causing a delay in its issue.

They presented the following report, which was adopted:

ORDER OF BUSINESS.

1. Reading the Minutes of the last Convention.
2. Admission of Members.

3. Report of Officers and Committees.
4. Election of Officers.
5. Retiring President's Address.
6. Induction of Officers elect.
7. Miscellaneous business.

All essays shall be read to open the discussions on the subjects to which they relate.

No member shall speak more than fifteen minutes, nor more than twice on the same subject without permission.

### I. ETIQUETTE.

1. Fraternal relations and courtesies among dentists and with physicians.
2. Intercourse with patients and the public relating to neighboring practitioners, and their operations.

### II. SURGICAL DENTISTRY.

1. Ulceration of the deciduous teeth, its effects upon the growth or health of the permanent ones.
2. Inflammatory diseases of the gums and periosteum, producing absorption of the sockets and loosening of the teeth; causes and treatment.
3. Bleaching teeth when discolored from loss of vitality; means for preventing their discoloration and ulceration.
4. Filling teeth and roots. What is gained by fang filling, and average of success?
5. The various plastic materials for filling teeth. Their relative or individual merits.
6. Hemorrhage after extracting teeth. Treatment.
7. Miscellaneous subjects relating to Surgical Dentistry.

### III. MECHANICAL DENTISTRY.

1. Surgical preparation of the mouth for artificial dentures. Should the roots of decayed and broken teeth always be removed?
2. When shall we insert pivot teeth?
3. The relative merits of the various materials used as a base for artificial dentures, and the method of mounting them.
4. Miscellaneous subjects relating to Mechanical Dentistry.

IV. UNFINISHED AND MISCELLANEOUS BUSINESS.

N. B.—The Executive Committee suggests that half an hour every morning be devoted to the presentation of models, improvements, and inventions, and the disposal of business not embraced in the regular order.

B. T. WHITNEY,<br>

F. SEARLE,<br>

WM. A. PEASE, } *Executive Committee.*

The Convention proceeded to the election of officers for the present year.

Dr. JOHN ALLEN, of New York City, was unanimously elected President.

On motion of Dr. Rogers, it was voted that in the afternoon session the members' names be called, and they take their seats on the side of the hall at the President's left.

Adjourned until three o'clock.

AFTERNOON SESSION.

The President called the meeting to order at three o'clock, and the balloting for officers was resumed, which resulted in the following selection:

*Vice-President*—Dr. J. D. WHITE, of Philadelphia, Pa.

*Recording Secretary*—Dr. F. SEARLE, Springfield, Mass.

*Corresponding Secretary*—Dr. B. T. WHITNEY, Buffalo, N. York.

*Treasurer*—Dr. JOHN T. METCALF, New Haven, Conn.

The retiring President, Dr. Buckingham, remarked that it was a source of regret to him to state that he had no address prepared to deliver on retiring from the Chair—his excuse was that, owing to the distracted state of the country, for some time there seemed to be an uncertainty whether or not the present meeting would be held, and since it had been so determined, it had been out of his power to prepare one. He was glad that two Secretaries had insisted upon this annual session's taking place, and now felt, from the number present, that his own fears were groundless; indeed, the more that he saw of this Convention, the more he was convinced of its value and its progress. The old American Association, where the whole work was done by a few, and the meetings were so controlled, was a failure; but an institution where all is free and open, with appropriate rules for government, he considered a necessity.

Drs. Rogers and Wetherbee were appointed a committee to conduct the President elect to the chair. Dr. Rogers said, "It is known that 'republics are ungrateful;' but surely this can not be true of the American Dental Convention, for they have called the man who stands first in mechanical dentistry in the country to preside over them. I introduce to you Dr. John Allen, of New York."

On taking the chair, Dr. A. expressed his thanks for the honor conferred, and his wish that it had fallen on some one more capable. He said since he was there, all he was or could do should be theirs. He had seen many changes in his life, yet none like the one now agitating the country. Still he could not but congratulate the Association upon the present state of the profession and the number in attendance, even considering the absence of many who usually were with us.

The other officers elect came forward and took their seats.

On motion of Dr. Wetherbee, it was voted that a reporter be employed to furnish the discussions for the daily papers published in New Haven. His object, he stated, was to carry this information outside of the dental journals, and, by giving it publicity, thereby acquaint the people with their designs, and interest them in the subject.

On motion of Dr. Hurd, it was voted that the Executive Committee make a report of the order of business and subjects for discussion previous to the final adjournment.

On motion of Dr. Wetherbee, it was voted that Dr. W. Hooker, of the Medical College, and other medical gentlemen of this city, be invited to seats upon this floor.

The President appointed the following as the Executive Committee: Drs. W. H. Atkinson, Cleveland, Ohio; George T. Barker, Philadelphia, Pa.; W. B. Roberts, New York City; I. J. Wetherbee, Boston, Mass.; Samuel Mallett, New Haven, Conn.

Dr. Franklin moved that the several dental journals be furnished with a copy of the Report of the Executive Committee, with the request that they will give said report an insertion in each number during the ensuing year. Adopted.

Dr. Wetherbee moved the appointment of a committee of three to revise the Constitution. After an amendment by Dr. Buckingham that the committee report before the final adjournment, the motion was carried.

The President called for essays or remarks upon the first subject for discussion, Etiquette, considered—1st, in "Fraternal relations and courtesies among dentists and with physicians;" and 2d, in "Intercourse with patients and the public relating to neighboring practitioners and their operations."

Dr. WHITNEY thought this one of the most important subjects for the consideration of the dentist, and yet it received but little attention even in the dental periodicals of the day. The practical or manipulative department may be found in books, but this could not be so learned. He referred to the difference to-day in the dental profession and what it was twenty years since, when he entered its portals; then each member wore his mantle of secrecy, and what knowledge he possessed was securely locked in his bosom. It was our duty, he thought, to impart all useful information that may tend to the benefit of others. Even as a matter of policy, it is best; for, by a narrow-minded course, we not only lose our own self-respect, but expose ourselves to being considered charlatans and quacks. We also owe much to the medical profession, to whom we have the claim of kindred; and particularly to the medical practitioner do we owe courtesy, and should cultivate with him the most friendly feelings, as there is often a mutual dependence necessary between the two professions.

Dr. ATKINSON thought the whole of etiquette was a personal matter, and yet every man of experience knows how sore a point it has always been. Every honest young man who enters the profession, and all are such at their commencement, asks, "What do I owe in respect to my elders?" and justly inquires, "What is etiquette?" Does he always see it in his elders? Do we even know exactly what it is? Does not every individual put his own construction upon it, varying from it according to the influences surrounding him? The golden rule, he considered, was all-sufficient, and is the only sure guide, in all cases; ask yourself the question, when in doubt, would I have others so act to me? The scientific man too often locks what knowledge he has gained in his own breast, and resolves that it shall die with him; but this is not just or right, for we must acknowledge that all we have comes from the great Father for the benefit of the race. Until these general principles are recognized, etiquette is useless. Earnestness also is essential to insure success, and if accompanied with honesty, few would fail. Even an old, hardened sinner can be awakened to his condition if approached with simplicity and honesty.

On motion of Dr. Mallett, Dr. Worthington Hooker was invited to address the Convention.

Dr. HOOKER referred to the pleasant intercourse which existed between the two professions, and the frequent necessity of meeting each other in consultation ; indeed he had that day been engaged with Dr. Mallet in the administration of ether as an anæsthetic. He did not consider the golden rule would in all cases answer in this matter of etiquette, but believed that errors were often made from misconceptions in both professions. He would instance such a case: A patient may present himself to the physician, suffering from neuralgia. Medical men, as a class, are too apt to ignore the influence which the teeth may exert, and will probably say the disease is due to some other cause. If, subsequently, the dentist is visited, he will probably say that the decayed teeth are the cause. In this expression of opinion the dentist does wrong, for he should first ascertain whether a physician has been consulted, and then advise with him ; and the same rule should be observed on the part of the physician. In this way a pleasant and high-toned intercourse will be observed, and the rights of both be maintained. Mere thoughtlessness was too often attributed to a want of courtesy. Much harm was done by men in all professions who profess to know everything, and yet expose nothing but their ignorance. He went to such a one in dental practice some time since, who perfectly flooded him with knowledge, and protesting he could determine the effect of ether upon any person at sight ; he would not be understood, however, as being opposed to knowledge, but would have all know as much as possible ; but let us not forget that others may be as well informed as ourselves. Dr. H., at the request of the Convention, gave at some length his views upon the proper exhibition of ether, and considered that for dental practice it was not necessary to produce total insensibility, but believed there was a period when partial ænesthesia is induced, in which sensation is lost without a loss of consciousness.

Dr. WETHERBEE said medical men often felt regret at the expression of opinion by dentists on diseases which did not belong to the dental profession ; thus presenting two conflicting judgments to patients, often tending to the disadvantage of both parties. We ought not to be jealous of each other ; but a relationship should exist, which should be firmly cemented with a just acknowledgment of the rights of both.

He longed for the time when dentistry would be more fully acknowledged as a twin sister of the medical profession. They have claims upon us, and we upon them, which can not be thrown aside; but we must first make it apparent to the community, as well as to other professions, that we are masters of our art. In our judgments of the work of other practitioners, let us be guided by the highest sense of justice and honor. In many cases he considered silence the better part of valor, as there are often circumstances over which the operator can have no control, which may affect the quality of the work; if we thoughtlessly pass condemnation, we also may be condemned by others for the same failings. He thought, however, when teeth came under notice that had been destroyed by amalgam, osteoplastic, or other base materials, we should, without referring to the operator, advise the patient to have them removed, and for the future avoid their use, stating our reasons for such a judgment. Dr. W. alluded to several cases in which he had consulted with physicians; their intercourse terminating with the pleasantest results, and had ever found them ready to impart information to the honest inquirer.

On motion, adjourned to meet on Wednesday morning at half-past nine o'clock.

SECOND DAY—MORNING SESSION.

The minutes of previous day were read and approved.

The following communication was received and read:

College of Dentists, England, 5 Cavendish Square, London, July 9, 1861.

To Prof. T. L. Buckingham,

*Dear Sir:*—The subject of the proposed "World's Dental Convention" has been brought under the notice of the Council of this College, and we are requested to communicate to you, as President of the American Dental Convention for the current year, that in the event of the General Convention being held in London during the Great International Exhibition of 1862, the College of Dentistry will deem it an honor to welcome their professional brethren from America, whose enlightened labors have tended so much to secure for the profession of dental surgery a distinguished scientific position in their own country, and have proved a noble example to the dentists of other countries.

The Council had hoped that the College of Dentists might be made available for the great meetings of the Convention, but inasmuch as the profession in England is so divided, it appears desirable that *neutral ground* should be chosen for this purpose, that both sections of English dentists might unite to welcome their brethren from distant lands.

We have the honor to remain, dear sir, with much respect, your faithful servants,

(Signed) George Waite, M.R.E.S., *Pres.*

Samuel Lee Rymer, } *Hon. Secretaries.*

Anthony Hockley, }

On motion of Dr. Franklin, it was accepted and ordered to be incorporated in the minutes of the meeting.

On motion, a committee was appointed, consisting of Drs. Buckingham, Wetherbee, and McIlroy, to take into consideration the letter, advise any action they may think expedient, and report at to-morrow's session.

The discussion on "Professional Etiquette" was resumed.

Dr. Hurd, of Brooklyn, fully approved of what had been said yesterday, and believed all had no doubt been profited, but considered we had but crossed the threshold, as the subject was both extensive and far reaching, even beyond the precincts of our profession and that of the physician. It required us often to descend from our lofty eminences and consult with those we may consider our inferiors; but who of us has not imperfections? and even a bungler should receive consideration if he is honestly striving to elevate himself—then it is he is entitled to the respect of every member of the profession. True etiquette goes even further, and requires silence upon others' faults; even when justice would seem to demand exposure, it prevents those significant winks and nods, which often convey so much, and all public criticism upon a brother's *writing or spelling.* Selfishness and envy must be thrown aside before we can hope to attain perfection. I had rather, said he, bless my own soul by a good act toward an enemy than do him injustice by an ill-natured remark. (Dr. H.'s remarks were delivered in a most eloquent manner, and were, as they deserved to be, warmly applauded.—Rep.)

Dr. Searle referred to the time, which was not far distant, when medical men looked upon dentists as mere mechanical operators, and therefore denied them their confidence and respect. But now, those men are passing from the field of action and a younger and more liberal class are taking their positions; and upon these we can exert an influence for good which will result in a mutual benefit. Particularly upon the diseases of the teeth can we give them instruction, and he had always found that it had been kindly and generously received. He would, however, have dentists know and assert their rights, and where this was done, it would be both beneficial to patients and medical practitioners.

The President, Dr. ALLEN, requested Dr. White to take the chair, and proceeded to read a paper upon "*Causes which retard Dental Progress.* (This will be published in a future number.—ED. REG.)

The subject of "Professional Etiquette" was resumed.

Dr. JARVIS considered the subject of fundamental importance, and would be glad if we could lay down some rule by which our actions might be governed in all cases. The golden rule is one of great beauty, but thought there were circumstances in which it would not apply. It might be thought preposterous to think of superseding it, but would prefer to adopt the following: First, Kindness, uniform and never to be forgotten. Second, Justice though the heavens fall. He would instance a case where the golden rule should be laid aside for these. A person consults a physician as to the cause of pain in a tooth which has been previously filled; he expresses his opinion by stating that the pain is due to the filling; if, when the dentist sees the case, finding the filling perfect, that the pain is the result of a bruise, he would ask, if we should apply the golden rule, would say no! If there be blame, and justice to others requires us to show it, he would never hesitate to do so, accompanying it with all kindness, but yet with justice as a requirement of the profession.

Dr. STOCKTON passed a high eulogium upon the kindness he had received from the elder members of the dental profession.

On motion, the next subject for discussion was taken up, being "Ulceration of the Deciduous Teeth, its effects upon the growth or health of the permanent ones."

Dr. J. D. WHITE remarked that he supposed the question referred to the effect of ulcerated roots, and would answer, that he should not always consider them injurious. It is true that by the early loss of the temporary, an indirect influence may be exerted upon the permanent teeth. He considered the judgment of the operator could be the only guide for the treatment, as if the ulceration is extensive, it may do injury to the permanent, but if not so, thought no harm would result. The presence of the deciduous teeth are of the greatest necessity for the perfect development of the permanent set, and never extracts such teeth, except upon the direst necessity. It is the great fault of operators to yield too readily to the importunities of parents in removing such teeth—indeed it seemed to be an almost American trait to be anxious to get rid of the temporary teeth; as a consequence, we meet constantly with contracted maxillary arches, particularly of the superior jaw, the result of this meddling. He had been censured for this course, but believed it to be the true one, as the disease to these teeth will often pass off, leaving them to perform their natural functions in the animal economy. The old practice, viz., that of the removal of all diseased teeth, both permanent and deciduous, he had seen the folly of; indeed had often cut off the ulcerated roots of deciduous teeth, where they had been pressed from their sockets, causing irritation to the cheek, being held in by a small portion of gum, embracing the neck of the tooth; and remarked that all were doubtless aware the process of absorption of the fangs was arrested when they were thus ulcerated.

Dr. ATKINSON agreed with Dr. White, that we often distort the faces of the dear children by our haste to interfere where "an angel would not dare to tread." We should have a higher conception of molecular action, and should study more closely the influences governing absorption of the fangs of the temporary teeth. He knew that we extract too many deciduous teeth, while with our own children we search out the cavities of decay and fill them. If we allow these teeth to disintegrate and be lost before the permanent ones are ready to take their place, there will be a lack of development, for the necessary molecules can not be deposited unless there is the requisite amount of circulation. He described ulceration to be merely the effort of nature to remove the broken-down and disintegrated tissue about the apex of the fang, and thought Dr. White's method of amputating such fangs, when possible, was correct practice.

Dr. BUTLER considered amputation of roots quite as practicable as that of a finger. It would be well to examine and see to what ulceration is due; it is usually the result of the death of the pulp, and this we should prevent, when possible, by filling deciduous teeth. He had, on several occasions, cut off the palatine fang of permanent molar teeth, and such have been retained and performed good service.

Dr. WETHERBEE referred to parents bringing their children, with the desire that certain teeth should be extracted; he would advise them against such action; they would frequently go away, but again return, begging him to operate; in such cases he would peremptorily refuse, considering it the worst of malpractice. We should not fail to explain to the parent, and enlighten them as to our reasons. He usually recommends, for the removal of the fetid condition of such teeth, a daily syringing, sometimes with creosote, more frequently with paregoric; has also treated such teeth and filled them successfully.

Dr. PRIEST spoke of the necessity of filling the deciduous teeth, and thought it our duty to educate parents as to its importance.

Dr. WOOLWORTH agreed with Dr. Priest, and had noticed where the deciduous teeth were extracted early, the permanent ones would also come through too soon.

Dr. BUCKINGHAM said that ulceration was only one of the effects of inflammation, but considered that the influence exerted would depend very much upon the general health of the child; in a perfectly healthy one, as bad results would follow, but with the reverse, such systemic disturbances may be exerted, that it would be necessary to extract the tooth; therefore, the judgment of the operator could be the only guide. The circulation and nutrition of the permanent and that of the deciduous teeth were so independent that he considered (except in rare cases) no local influence could be exerted upon the former by the ulceration of the temporary teeth.

Dr. ATKINSON drew a diagram, and gave a lengthy and interesting description of the formation of an alveolar abscess; he would, under some circumstances, extract ulcerated deciduous teeth, when occurring at an early stage, when the permanent ones were in a soluble condition; considered such cases rare. In one case could recall, where ptyalism was induced in a child until sloughing of the process containing the deciduous teeth took place, and yet the person eventually had a good set of teeth. Dr. A. was asked and answered several questions on this subject.

Dr. White, in reply to a question, as under what circumstances he would extract deciduous teeth, said that individual cases prove nothing, but we must form our judgment from the great aggregate of practice. No surgeon, let the description of the case be ever so minute, would venture upon a prognosis without seeing the patient; and this answer he would make to the question; he must see the patient, or he could tell nothing. It was impossible to put on paper just what you would do in all cases; could only say, would extract the diseased deciduous ones, when he felt they were damaging the permanent set; would refer to the importance of taking notes of everything that occurred in practice; particularly should this be observed by young practitioners. Many things were to be taken into consideration before determining to extract, and where the general health seemed to be suffering, we should call in consultation a medical adviser.

Dr. Roberts would ask Dr. White, what proportion of teeth he extracts for children where he fears the permanent ones will be affected?

Dr. White seldom extracts for the effect upon the permanent set, but usually from the influence exerted upon the general health. Has seen much injury done by early extraction, but had charity to think the children might have been suffering from constitutional disturbances; but thought, if the permanent tooth is laid bare by early extraction, when the enamel is not dense and will absorb the fluids of the mouth, injury must follow.

The discussion, which was of an interesting character, though confined much to questions and answers, was continued at some length, being participated in by Drs. Franklin, Whitney, Palmer, and others.

Drs. Wetherbee, Whitney, and Rogers were appointed a committee to revise the Constitution.

Communications were received from Dr. Charles Hooker, inviting the Convention to visit the medical institution of Yale College; also from Mr. Herrick, extending an invitation to the members to visit the Trumbull Gallery, Museum, and the various college buildings.

On motion, the thanks of the Convention were tendered to these gentlemen, and the Secretary was instructed to communicate the same.

Adjourned to meet at three o'clock.

SECOND DAY—AFTERNOON SESSION.

The President called the Convention to order at three o'clock.

On motion, the second subject in "Surgical Dentistry" was passed over, and the third, "Bleaching Teeth, when discolored from loss of vitality; means for preventing their discoloration and ulceration," was taken up for discussion.

Dr. J. D. WHITE considered the term "dead tooth" an erroneous one, as even when the pulp dies, the periosteum still performs its limited function of nutrition; but this error of language was due to the fact of the deficiency of a good dental nomenclature. The discoloration seen in teeth, after the death of the pulp, is usually attributed to the breaking up of the red corpuscles of the blood, the hæmatine or coloring principle of the same permeating and becoming lodged in the tubular structure of the dentine. This he considered was not the cause, but thought it due to an absorption of the fluids of the mouth. Again, teeth will not discolor after they have been removed from the mouth. A blow or other injury will discolor a tooth; and, indeed, had seen teeth with live pulps that presented the red appearance. Why this was so he could not tell. A tooth may also become discolored by an inflammation of the pulp before its death. As to the bleaching of teeth, each one had his favorite method; he would only refer to one which had been spoken of in one of the recent dental journals, viz., Labarraque's solution; this he thought very deleterious, as a tooth immersed in it for twenty-four hours, though turned white, has its normal structure completely destroyed, and would rapidly discolor in the mouth.

Dr. BARKER regretted that he could not agree to all Dr. W. had said, that the coloration was not due to the permeation of the disintegrated red corpuscles; because teeth are not discolored out of the mouth after removal. In a healthy tooth, with a living pulp, the tubuli of the dentine are filled with the liquor sanguinis of the blood, which conveys to those tissues the materials necessary for their proper nutrition; this circulation is due to the ever-present action of exosmose and endosmose, and these continue after the death and disintegration of the pulp; and the broken-down, red corpuscles, which previously were too large to pass into the open-mouthed dental tubuli, may now by endosmodic action enter, thus inducing discoloration; but after removal from the mouth, this action would cease. In bleaching, the first duty should be to bring the tooth to as healthy a condition as possible, before attempting to restore the color. That teeth may be nearly restored to a natural color he did not doubt, as the experiment had been tried on one of his own teeth, which had been discolored for four years, accompanied with a slight chronic periodontis; his friend, after removing the gold filling in the pulp cavity, treated the tooth and restored it to a good color by using a preparation of prepared chalk and chloride of lime, introducing it each day.

Dr. ATKINSON said that the subject of bleaching required a vast deal of knowledge of the laws of chemical equivalency. Color is not an entity, but an arrested fractional part of light. To bleach a tooth, the whole decayed mass, hard or soft, must be removed. Chlorine is the great bleaching agent, and when used, it must be so combined as to produce its least deleterious effects. For this purpose he used the *undeliquiesced* chloride of zinc, crowded into the cavity of the tooth.

Dr. BARKER asked Dr. A. if chloride of zinc was a bleaching agent, or had such properties.

Dr. ATKINSON replied that he was misunderstood; that he expressly stated that chlorine was the bleaching agent; and that the chloride of zinc, when introduced into the tooth, *undeliquiesced*, absorbed water from the mouth, becoming liquid, and thereby the chlorine was liberated to effect its bleaching.

On motion, Prof. G. F. BARKER was requested to give his views on the chemistry of the subject of bleaching. He stated that while it is true that chlorine is the great bleacher, it is quite doubtful whether this action is due to the chlorine itself, since it will not bleach except in presence of moisture. In this case, where water is present, the chlorine unites with the hydrogen, setting free the oxygen, which in a nascent state, as ozone, perhaps, effects the bleaching. Moreover, the active agent in both the chloride of lime and soda (the last Labarraque's solution) is not chlorine, as such, but hypochlorous acid, which acid is set free from combination by any other acid, even the carbonic from the atmospheric. In the chloride of zinc—which is a *binary* compound, while the others named are *ternary*—no such action can take place. And he contended that the simple deliquiescence of this salt—its passage from a solid to a liquid state—could not, in the nature of things, decompose the salt and set free the chlorine for bleaching. "This is not my opinion," he concluded, "but the fact. I have no *opinion* on the subject."

Dr. BUTLER asked to what the influence of chloride of zinc was owing, as he had noticed its beneficial effects in whitening teeth.

Prof. BARKER replied that whatever virtue there was in this substance to whiten, was due rather to the salt as a whole, than to either of its constituents. His impression was that it acted physically in presenting a white ground in the tooth, and perhaps assisted to soften and remove animal coloring matters.

Dr. HAYES said that after trying all the so-called "bleachers," he had returned to the old practice of scraping the decayed and discolored bone from the pulp cavity and canal as much as possible, and then filling solidly with gold, and obtained good results.

Dr. WETHERBEE had for ten years discarded bleaching agents in the treatment of discolored teeth. He first removes all foreign matter in the pulp chamber, and then thoroughly scrapes the inside of the fang, cutting away all dead bone, then fills the tooth solidly with gold. Teeth well filled will often return to a nearly natural color.

A discussion took place between Drs. Wetherbee and Atkinson as to the anatomical structure of the tooth, and the existence of cementum in its interior. Dr. A. contended there was no internal cementum, but only an exterior, covering the fang above the neck of the tooth; while Dr. Wetherbee cited Fox and Hunter to prove that it was also found internally, acting as a cushion to the dental pulp.

Dr. J. D. WHITE stated that from drawings made from actual specimens when he was microscopist to the old American Dental Society, the structure of a central incisor was bivalvular, resembling a clam; there being two thin plates of enamel lined with dentine, having a membrane on its interior surface, and a large cavity filled with pulp. There is a crack along the cutting edge of every tooth. To prove that this was not accidental, he examined teeth before their protrusion through the gum, and found it true of these.

The tooth from which the microscopical specimen was taken was from a dead subject; it being one that had never passed through the gum.

Dr. BARKER said he would like to refer to the subject of internal cementum, which had called forth remarks from Drs.

Wetherbee and Atkinson. He would commence by drawing a diagram of a tooth, showing the three different tissues, as described by the best physiologists and microscopists of the day, and would ask why dentine or enamel could not be denominated cementum? and would answer by stating that, first, each one of these differed in the quantity of organic and inorganic material; second, they each were differently constructed, anatomically and physiologically, also presenting quite opposite appearances under the field of the microscope. Cementum has been justly denominated tooth-bone, as it nearly approaches in structure true osseous tissue. If a section of bone be examined with the microscope, we see what are denominated Haversian canals, with branching canaliculi and lacunæ—their use being to convey nutrition to the part which is always extra-vascular—and if a section of cementum be used, we shall see but a modification of this structure, and would ask if any one had ever seen such an appearance from an internal section of a tooth, and doubted if such could ever be obtained. Secondary or osteo dentine under the microscope will present none of these characteristics.

Dr. WETHERBEE said, he would refer Dr. Barker to the works of Drs. Fox and Hunter, to prove the existence of internal cementum.

Dr. BARKER replied that he would refer Dr. W. to the works of John Tomes, who commenced his investigations where the others ended.

Dr. WHITE stated that the best practice to whiten the teeth was to open and thoroughly clean them, keeping them dry and open to the air and light. This would be better than bleaching, as he had known injury to result from the use of chemicals.

Dr. PALMER had abandoned bleaching agents, but cleaned out the tooth well, and if very much discolored, took out all tissue that was disintegrated, then put in some white lint or flax and filled it solidly with gold, and thus gets a good color.

The Committee on Constitution made a report, through their chairman, which was adopted, and the committee thanked for their promptness.

We quote only the following from the Constitution:

"ART. 8. Any dentist may become a member of this Convention on the conditions herein mentioned, unless objection is made, in which case he may be excluded by a vote of the Convention.

"ART. 9. Honorary members may be admitted by a special vote of the Convention."

Adjourned to meet on Thursday at 9 A. M.

### THIRD DAY—MORNING SESSION.

The Convention assembled and considered various dental inventions for an hour.

Dr. Franklin presented to notice a new style of vulcanite teeth, invented and manufactured by Mr. S. S. White, of Philadelphia. The peculiarity consists in having a protuberance molded upon the block to assist the pins in retaining the teeth in position in the rubber base. He also exhibited an improved vulcanizer, with a fusible metal gauge as his own invention.

Drs. Hayes and Whitney also presented their own improvements in vulcanizers.

The committee to whom was referred the London communication made a report, which, after considerable discussion, was recommitted—Drs. Hayes, Rogers, and Franklin being added to the committee.

On motion, the Convention went into ballot to select the next place of meeting. After a long time spent in deliberation, Trenton Falls, New York, was determined upon, the Convention to assemble there on the second Tuesday in August, 1862.

The Executive Committee reported the following as the order of business for the next meeting:

1st. Admission of Members.
2d. Reading Minutes of the last Convention.
3d. Report of Officers and Committees.
4th. Election of Officers.
5th. Retiring President's Address.
6th. Induction of Officers.

All essays shall be read to open the discussion on the subjects to which they relate.

No member shall speak more than ten minutes, nor more than twice on the same subject without permission.

### I. MISCELLANEOUS SUBJECTS.

1. Anæsthetics. Their use and relative value.
2. Alveolar abscess.
3. The causes influencing an abnormal development of the teeth.

II. OPERATIVE DENTISTRY.

1. Filling Teeth. Simple and complicated cavities.
2. The Dental Pulp. Its varied treatment.
3. The Extraction of Teeth.

III. MECHANICAL DENTISTRY.

1. Artificial Dentures, temporary and permanent.

IV. UNFINISHED BUSINESS.

N. B. The Executive Committee suggest that half an hour every morning be devoted to the presentation of models, improvements, and inventions, and the disposal of business not embodied in the regular order.

On motion, Dr. Atkinson was requested to read his essays, which he proceeded to do—the first one being entitled "What lack I yet?" second, "Life." Both of these papers will appear in the October number of the Dental Cosmos.

Adjourned to meet at three o'clock.

THIRD DAY—AFTERNOON SESSION.

The President called the Convention to order at 3 o'clock.

An invitation was received from Prof. B. Silliman, jr., for the gentlemen of the Convention to visit the buildings and laboratory of the Sheffield Scientific School. Accepted, and the thanks of the Convention returned.

Dr. Perine moved that before the meeting adjourned, a certain time be allotted to discuss the propriety of appointing dentists in the army and navy.

The next subject in order—"Filling Teeth and Roots; what is gained by fang filling, and average of success"—was then taken up for discussion.

Dr. ASAY described the method employed by him, differing not materially from the usual one.

On motion, the time for each speaker was reduced to ten minutes.

Dr. J. D. WHITE, in answer to the question as to what is gained by fang filling, said that we gain a great deal, in so far as metal well packed will shut out the gases and fluids which decompose and injure the structure of the teeth, also closing a cavity that may become filled with pus. The assertion that the pulp cavity is empty after the destruction and removal of the pulp is erroneous; this can be proven by the use of a silver probe, which will always be blackened. Again we see the frequent discoloration of the whole body of the tooth. Softening of the tooth structures takes place in all cases after the removal of the pulp, but this is much greater when the fangs are left unfilled. Does not fill immediately after the pulp has been extirpated, but waits until anastomosis takes place between the divided blood-vessels of the pulp and those of the periosteum, then plugs up the root and crown tightly. Regarded it best so to fill fangs that it may be removed in case of trouble, allowing the proper treatment to be extended. Dr. W. also gave his method of treatment of alveolar abscess.

Dr. Barker said that, in considering this subject, he would take, for an example, a tooth where the pulp had been destroyed and removed, and would ask, what would be the nature of the inflammation that would ensue at the apex of the fang, and might not the higher grades be induced, which could not be properly treated if the tooth was immediately filled? His custom was to fill with cotton, leaving it for one week after the extirpation of the pulp.

Dr. Wetherbee thought fang filling the most important work, as it was the best evidence of the ability of the dentist. He would take issue with the last speaker, as to the propriety of not filling immediately. His own practice was to remove the pulp, and having secured the cessation of bleeding by applying creosote, fills at once, and would not be hired to delay it for a single day. Success had taught him that his method was correct, for in no case, during the past ten years, had he seen a single unfavorable case, but had seen bad results where the tooth had been left after the removal of the pulp. Referred to several interesting cases in his own practice, showing the advantage of immediate filling.

Dr. Perine considered that to obtain success we must adopt an eclectic practice. Every one, no matter what his capabilities may be, must, in a certain number of cases, meet with failures. He referred to a case where he treated two teeth, which, after a short time, proved unsuccessful; he removed the fillings, treated the teeth and then filled, but with the same result. Our register may state success after success, but we have no means of knowing how many unfavorable cases fall into the hands of other practitioners; they lost far more than they were aware of, even where the teeth were well filled.

Dr. Roberts believed when a tooth is diseased, the result of the death of the pulp, it will always remain so, and though it may remain for a long time in the mouth, there will always be more or less soreness felt in it, not enough, perhaps, to annoy the patient, but yet an unnatural feeling. After destroying and removing the pulp, when we fill the fangs, it should be done solidly to the apex; but in the vast majority of cases, we fail to do this, even where we honestly think we do it; particularly is this so in the molar teeth. The manner of filling in many cases must be varied, as certain circumstances and conditions of the patient must be taken into consideration. We may hammer and work at some teeth as much as we like without causing the least disturbance, but in others the most gentle treatment will produce unpleasant results. He did not believe every tooth could be saved, and thought the best operators may fail, from circumstances beyond their control.

Dr. Tefft related a case of fang filling in his own practice —he would ask Dr. White if those having vitiated constitutions were not the hardest patients to treat. Dr. W. replied that he was always governed by the diathesis and temperament of his patient, and had some for whom he extracts teeth immediately on the exposure of the pulp.

Dr. Metcalf referred to an interesting case that terminated in ulceration of a malignant type; he extracted the tooth with the socket attached. He attempted also to treat an exposed pulp in another tooth, but the same result supervening, he of course failed. The patient was of a scrofulous diathesis.

Dr. Franklin said, the failures which are reported are not unfrequently the result of a misunderstanding of the case by the operator; in his opinion, if a definite line of practice was pursued, we could obtain more nearly constant results.

Dr. Atkinson gave, at considerable length, his views as to the change which takes place at the apex of the fang after the pulp has been extirpated, illustrating them by means of a diagram. He thought there was a misapprehension in the minds of many, and that we often go farther than we should, thinking we know more than we do. Thought, if there was no inflammation about the fang, it could be filled immediately —first extirpate the pulp, cleaning out well the pulp cavity, and then proceed to fill. If alveolar abscess is induced, don't take out the filling, if it is a good one, but treat it from the outside, by drilling through the process.

Dr. SEARLE said, we often had to be governed by the circumstances of the patient, as many of his own came from the country, and he could not see them frequently. In cases where he opens into a tooth with a dead pulp, he dares not fill immndiately, particularly if he can not control the case; therefore puts in a test filling, and tells the patient in case of trouble to visit the nearest intelligent practitioner, and let him see the case. He never dares to fill a tooth so that he can not remove the fang filling in case of inflammation; also prefers to open into the pulp chamber from the palatine surface of front teeth, in preference to opening through the cavity of decay.

Dr. WHITE explained his manner of rolling a piece of gold so as to be easily introduced and removed from the fangs of teeth.

Dr. BURRAS read a paper on "*Mastication and Articulation of Artificial Dentures.*" (For this see future number of the Register.—ED )

Drs. Atkinson, Perine, and Franklin were appointed a committee upon the question of appointing dentists in the navy and army.

The committee on the London correspondence reported the following letter in answer:

NEW HAVEN, Ct., Aug. 8th, 1861.

Messrs. WAITE, RYMER, and HOCKLEY,<br>College of Dentists, London,

GENTLEMEN:—The undersigned, a committee of the American Dental Convention, appointed to reply to your letter addressed to this body, beg leave respectfully to say that the fraternal and cordial spirit of your communication has afforded the Convention the most sincere pleasure, and inspired its members with renewed, and, if possible, higher regard for the professional brethren in England.

The committee are requested to return grateful thanks for the very kind manner in which you have been pleased to tender a welcome to American dentists at the proposed General Convention in 1862, and will be happy to make such response as the circumstances of the future shall allaw.

With the highest esteem, gentlemen, for yourselves personally, and for the distinguished body you represent, we have the honor to subscribe ourselves very faithfully and gratefully yours,

(Signed) T. L. BUCKINGHAM,
Ch'm. Com. Am. Dental Association.

On motion of Dr. Franklin, it was

*Resolved*, That the officers of the American Dental Convention extend a cordial invitation to the dentists throughout the United States, to attend the World's Dental Convention, to be held in London, in 1862.

On motion, adjourned to Friday at 9 A. M.

FOURTH DAY—MORNING SESSION.

The President called the Convention to order at 9 o'clock, the usual half hour being devoted to the consideration of inventions.

Dr. Hill spoke of the advantage of the material known as "Hill's Stopping," and thought no dentist could afford to be without it, as it was invaluable as a temporary filling; and had so perfected it, that it was impermeable and water-tight; also showed the method of properly introducing it.

Dr. Asay presented his method of attaching teeth to metallic plates by means of hard rubber.

Dr. Searle presented an approved method of recording dental operations.

The committee on "Appointing Dentists in the Army and Navy" reported that the subject appeared to them of so much importance that it was necessary that more time should be given to consider it than the present session would admit of.

On motion, the committee was continued with discretionary powers, and Drs. J. D. White and I. J. Wetherbee were added to the committee.

The next subject in order was taken up, being "The various plastic materials for filling teeth: their relative or individual merits."

Dr. WHITE referred to the article published in the August number of the Dental Cosmos for 1861, upon the oxychloride of zinc, as the merits of the material were honestly, though modestly enumerated. He was, at first, opposed to the use of these materials, but Dr. Metcalf had sent to him a small quantity, which he had used. In the first case, the filling, after being in eighteen months, continued sound, there being no permeation, shrinking or cracking; in another case, where a tooth had been filled with gold without arresting decay, the oxychloride was introduced, and the patient thought the tooth felt more comfortable, and the decay did not continue. Whenever a tooth can be filled with gold successfully, will always use it to the exclusion of other materials. A time was when every one was disgraced who touched anything but gold, because so many had been deceived, both patients and practitioners; and he was glad that time had passed, and that there was now a disposition on the part of the profession to give all materials a fair trial. When some professor of chemistry said the oxychloride would destroy the enamel and the dentine, he took a tooth and filled it, and on examining it a month afterward, found there was not the slightest change in the dentine upon the walls of the cavity.

Dr. WETHERBEE said, his faith was not as large as Dr. White's, for the material had received with him a severe blow, and justice to the profession required him to present the serious objections to its use. He referred to a case, where a dentist had introduced it in five teeth of fair quality, stating that it was better than gold, as this would not expand or contract as gold would do, on taking hot or cold articles in the mouth. After it had been in the teeth six months, he saw the case, and found three of the fillings were out, and one nearly so, while the teeth were sensitive; the decay being quite deep. The remaining one was left in, and at the end of three months it came out, leaving the cavity three times the original size. He assured the lady the material should never have been introduced, and was satisfied the osteoplastic had affected the dentine; he subsequently filled the teeth with gold. The article could not be made so that it would not absorb water, and therefore defied any one to make a water-tight filling; and had repeatedly reddened litmus-paper, and even the writing paper in which it was rolled was discolored from its acid.

Dr. METCALF asked Dr. W. to what substance in the material he attributed the effect upon the dentine?

Dr. WETHERBEE replied, he thought it due to the *oxide of zinc.*

Dr. METCALF said that, so far as his knowledge of chemistry went, oxide of zinc exerted no action upon the dental structures; about the chloride he was not so positive, but when these were combined, as in the oxychloride, the mixture was perfectly harmless. This was also the view of Prof. Silliman, to whom he had applied when first manufacturing the article. If it was properly made, it would not absorb water; and though he did not consider it would take the place of gold, thought, in some cases, it would be of use. The practice of a dentist should be "eclectic," as what may be invaluable in one case, may be useless in another.

Dr. WHITNEY asked Prof. Barker for some light upon the chemistry of these substances.

Prof. BARKER replied that whatever of knowledge he possessed on the subject was theoretical more than practical. From the mode of manufacture given in the Dental Cosmos, he should doubt very much such a thorough combination of the oxide and chloride in the manner of preparation indicated, as would entitle the substance to the name "oxychloride." The oxide of zinc is perfectly inert, and would not be injurious as a filling. The chloride is an active agent, zinc hardly neutralizing the chlorine more than did hydrogen. Chloride of zinc is the material used as a soldering fluid by braziers, to dissolve the scale of oxide and render the metal bright for the union of the solder. Judging from its active properties, he thought its action would be decidedly injurious to teeth. From Dr. Wetherbee's statement that the osteoplastic had an acid reaction, he inferred that the mixture was not made in just the proportions to insure no excess of its components.

Dr. WHITE said, he was probably the first in this country to use chloride of zinc. He placed a tooth in a solution of chloride of zinc for three years, and he thought it was rather improved when it came out, though it was sound enough before. If the chloride removes the animal matter, this is what we want to get at. He used to be told that sugar was bad for the teeth, but since he grew older he had seen its falsity, and even had incorporated it in a dentifrice, for the sake of its grit.

Prof. BARKER stated that in justice to Dr. Metcalf, he ought to state that oxychloride of zinc, if properly made, would not, in his opinion, injure the teeth. He said that he supposed the action of sugar was due to its fermentation, whereby it became converted into acetic acid, which would act on the lime salts of the bone. The relative hardness of enamel, dentine, cementum, etc., would cause the action of carrosive agents to vary immensely.

Dr. MALLETT entered his protest against its use.

Dr. STEPHENS thought it useful, if it would save teeth that could not otherwise be saved; but too much should not be claimed for it; too many in the community held it up as better than gold for filling. Dr. Metcalf was too honest to offer any article which was not in his hands valuable.

Dr. Smith, of New Haven, uses it when he thinks he can save a tooth with it, that could not be filled with gold. He considered Dr. Metcalf's preparation the best one, and had used it with good results.

Dr. Roberts believed it to be of value where teeth were exceedingly frail, not of course equal to gold, but its advantage was that it could be introduced where gold could not. It was the duty of the dentist to use what was the best material for the individual case, and his judgment must determine that. He related a case in his own mouth, where a gold filling was unsuccessful, and a bone filling subsequently introduced perfectly preserved the tooth.

Dr. Hayes asked Prof. Barker if a decomposition could not take place between the oxychloride of zinc and the phosphate of lime in the teeth.

Prof. Barker replied that we have no evidence that such a change did occur. But even if it did, the interchange of elements would break up the arrangement, and so disintegrate the tooth.

Dr. Buckingham had experimented with the different plastic materials, and found them composed of nearly the same chemical constituents. The great difficulty in manufacturing it was to obtain a uniform result, sometimes producing a substance as hard as a stone, and in others it would fail to harden, owing to impurities in the oxide of zinc; again, if the chloride be in excess, it will be constantly tasted, the animal matter of the tooth will be destroyed, and the filling will fail. The fluids of the mouth vary, and in some cases will be acted upon more than others. He, therefore, in view of these objections, preferred to use some other material, though he would take issue with Dr. Wetherbee as to his objections, which might as well be urged against arsenic and numberless other articles of the U. S. Pharmacopœia; indeed the objection had been urged to arsenic being placed there because its indiscriminate use would be disadvantageous. He had used the oxychloride as a temporary filling to remove the sensitiveness of dentine with good results, leaving it in from one day to two weeks.

The next subject was then taken up, being "Hemorrhage after Extracting Teeth, and its Treatment."

Dr. Atkinson referred to the invaluable styptic which has lately been introduced, viz., persulphate of iron, with which the most obstinate cases of bleeding can be easily controlled.

Drs. Burras, Whitney, Buckingham, Barker, Asay, Wetherbee, Roberts, and Hill gave their methods of treatment.

On miscellaneous subjects, relating to surgical dentistry, Dr. FRANKLIN read an amusing pcetical paper, on the advantages of artificial dentures after the failure of operations to save the teeth.

Dr. WHITE, in answer to a question, said he had never met with a case of discoloration which he thought due to the use of arsenic, and did not fear to use it in cases of sensitive dentine. At one time, he would admit, his mind was trammeled with theory, but that time had passed by. He was free to confess he could not get along without using arsenic, and did not see how others could do so. The various theories that had been introduced failed to account for the sensitiveness of dentine, but thought it due to the presence of nerve fibres in the tubuli. For twenty years he had been practicing where the use of arsenious acid was condemned for destroying sensitiveness; uses it dry (after having been thoroughly triturated in a mortar) in the cavity, keeping it in with cotton or wax. The delicate nerves which have been seen bv Mr. Tomes, and by him described, are destroyed, but the bone is not acted upon. True, he might sometimes kill a pulp, but would say that he saved a great many that otherwise would have died. In the approximal cavities of front teeth does not use it, but uses creosote or chloride of zinc—never uses creosote and arsenic together.

Dr ATKINSON said that when dry arsenic is put in a cavity, it will become a solution by the affinity which exists between it and some of the chemical equivalents of the tooth. He thought, from his own investigations, and also from those of others, that no nerve fibres could be found in the tubuli. Sharp, quick cuts with an excavator will often relieve the sensitiveness, though he uses undissolved iodine, and sometimes a covering of Hill's stopping. Referred to the importance of having pure creosote, a good test for which is olive oil, dissolving without leaving a precipitate, and said he never filled any cavity without first having wiped it out with creosote.

Dr. BUTLER said, after using arsenic, either for destroying a pulp or for sensitive dentine, always uses a solution of iodine, leaving it in the tooth for a few hours. The fluids of the mouth will remove the discoloration, the iodine will also relieve the irritation, and neutralize the effect of the arsenic.

Adjourned to meet at three o'clock.

FOURTH DAY—AFTERNOON SESSION.

At the appointed hour the Convention came to order.

The next subject in the order of business was taken up,—"Surgical preparation of the mouth for Artificial Dentures; should the roots of broken and decayed teeth always be removed?"

Dr. BUTLER instanced several cases where he would not extract roots of broken front teeth, but preferred to fill and leave them, that natural expression of the face might be preserved, and absorption be prevented.

Dr. ROBERTS considered the question could be answered both by yes and no, and thought the word "always" should have been stricken from the question. In preparing the superior arch for an artificial denture, would always extract all roots; but would not, in every case, in the inferior one. He instanced cases where he would prefer to fill the root rather than to remove it.

Dr. ATKINSON said, experience was the best teacher, and his own had taught him that it he wishes to retain the natural expression of the mouth, the fangs had better be left, if they are perfect, down to the edge of the alveolar process. The points of insertion of the muscles are also retained, which is of great importance. When nature in her efforts pushes out these fangs, he cuts them off even with the process; they were also valuable in equalizing the pressure of the artificial plate.

Dr. SUTTON considered there were cases where it would be best not to extract fangs; where, for instance, there was a short lip, and considerable gum was exposed, the fangs, if left, would contribute to a better expression.

Dr. HAYES related a case where he left two roots in the superior arch—the patient refusing to have them removed—over which he placed a set of teeth; it had been worn for twelve years successfully, and he kept cutting them off from time to time.

The next question, "When shall we insert Pivot Teeth?" was passed over, Dr. FRANKLIN only stating it should be done when we have a perfectly sound root, and the operation can be performed satisfactorily.

The next subject, "The relative merits of the various materials used as a base for artificial dentures, and the method of mounting them," was taken up for discussion.

Dr. WHITNEY said, his experience had been formed by many years' use of silver, gold, continuous gum, and vulcanite work. Of all these he preferred the last named, thinking it superior to anything else. If the impression is perfect, we could be sure of a fit in every instance. The twang, which was so much of an objection in other materials, was in this obviated, besides the cost was not so much to the patient, and it yielded as large a profit as other work to the dentist.

Dr. PARMELE said, he had worn the rubber work, and it produced in his mouth a nasty, sour and sickening effect; the same experience was produced in the mouth of one of his own patients. He did not consider vulcanite to be equal to either gold or silver—indeed thought it valueless.

Dr. ROBERTS said, all were not constituted alike and we should only use what was the best for the individual case. His own experience had taught him nothing was equal to Allen's continuous gum work, when properly made.

Dr. MALLETT said, he had been the first to experiment with rubber for artificial dentures in the United States, having perfected it and used it for the last six years. This was a year before Dr. Putnam, of New York, began, and he, therefore, had no claim to the invention. He also referred to the claims of other "so-called inventors." The longer he used the vulcanite work, the better he was pleased with it, and this was also the testimony of his patients, many of whom had worn other materials.

Dr. HILL corroborated the facts in reference to the first use of rubber work by Dr. Mallett, he having seen many of the experiments of that gentleman. He believed it to be a very valuable article, but thinks continuous gum work the most beautiful, and the nearest to perfection.

Dr. BUCKINGHAM said that Dr. T. W. Evans, of Paris, claimed to have been the first to vulcanize rubber plates, and to have manufactured the first set of teeth for Mr. C. Goodyear.

Dr. ALLEN, at the request of the Convention, gave at some length his method of manipulating; presenting many valuable and interesting hints necessary to success with the continuous gum, and closed his remarks by stating his reasons for a preference to continuous gum work; his confidence increased the longer he used it, and had no difficulty in obtaining perfect fits.

Dr. Franklin said, the relative value of different kinds of work can only be determined by the amount of good that can be accomplished. His own preference was for vulcanite, and time had proven its purity and indestructibility. The vulcanite work had the same difficulties to contend with in the beginning that continuous gum work had, viz., from the worthless gum palmed off on the profession and public, and the ignorance of the proper vulcanization. The object of many was to vulcanize in too short a time, which, at the high heat necessary, would spoil the gum. Thought the adaptation of plates excelled all others, though care was necessary to attain perfect results.

Dr. White said, not having had time to experiment with it himself, he had concluded to wait until the article had been perfected, but for the last two or three years had thought favorably of it. The great difficulty, in all these materials, is, that the inventors claim too much for them.

Dr. Atkinson said, we all have a prejudice against new things, but that the statistics of success could be the only sure guide to determine which was the best; therefore, as these cannot be obtained, we shall still be in the dark. For entire dentures, he considered the continuous gum on a platina base unparalleled. In the vast majority of cases, vulcanite, its price considered, stands as unparalleled. For under sets, he thought nothing so good as rubber. For upper partial sets, he preferred gold. For entire sets, with "plumpers," as they are termed, he preferred continuous gum, and next rubber.

Dr. Allen said, platina plates, after being struck up, should be placed in acid before being subjected to heat. If this is not done, the foreign metals, which have incorporated themselves in the platina, will give it a rough and dark appearance.

Dr. Searle referred to an interesting case, where he could get no adhesion of the plate to the roof of the mouth. Succeeded in so doing by fitting an oiled silk valve to the air chamber.

Miscellaneous subjects being taken up, a discussion ensued, whether the weight of a set of teeth was a disadvantage. Drs. White, Burras, Allen and Franklin concurred in saying that it was not a drawback.

Dr. Butler referred to the importance of properly selecting teeth.

Dr. Crossett, at the request of many members, addressed the Convention. He said he came from California to receive, rather than impart instruction, and had derived benefit sufficient to repay him for coming. If agreeable, he would give a short description of the progress of dentistry in his own State. When he went there, the profession generally were using quartz fillings and other materials where gold should have been introduced, but now a better practice was being adopted, and all base articles were confined to their appropriate places. There exists at this time a prejudice against the use of vulcanite, and but two individuals use continuous gum work.

On motion, a vote of thanks was tendered to Prof. Barker for the interest he had added to the session. Also to the press of New Haven, and especially the *Journal and Courier*, which gave an interesting report of each session.

The hour (six o'clock) having arrived for which the hall was rented to other parties, the President, after a few congratulatory remarks, put the question for final adjournment, and with seeming reluctance the Convention adjourned *sine die.*

It would be unjust were we not to state that our report fails to do credit to many of the most interesting discussions of the Convention, several of which were of a conversational character, and could not be reported. The uniform attendance and interest on the part of the members, and particularly of the ladies, was deserving of great praise, and contributed much to its happy character. The place selected (New Haven) was, we think, unexceptionably the very best that could have been chosen, as the kindness received by the members of the Convention, both from those of our own and other professions, will attest.—*Dental Cosmos.*

